# Decreased Mortality in Patients With Severe Bronchospasm Associated With SARS-CoV-2: An Alternative to Invasive Mechanical Ventilation

**DOI:** 10.7759/cureus.10822

**Published:** 2020-10-06

**Authors:** Rafael Salazar, Alejandro Hallo, Sebastian Vasquez, Steffy Reinthaller, Juan Echeverria

**Affiliations:** 1 Emergency Department, Hospital Pablo Arturo Suárez, Quito, ECU; 2 Internal Medicine, Hospital de Especialidad Eugenio Espejo, Quito, ECU

**Keywords:** covid-19 outbreak, covid-19 management, invasive mechanical ventilation

## Abstract

The number of patients with acute episodes of severe bronchospasm needing intubation and ventilatory support has increased rapidly during the severe acute respiratory syndrome coronavirus 2 (SARS-CoV-2) coronavirus disease 2019 (COVID-19) pandemic. Although medical consensus upholds the use of ventilatory support in this pathology, its survival benefits remain unclear. To improve the outcomes and survival rates, a bundle of early respiratory therapy with a pharmacological rescue regimen was provided to four patients with bronchospasm secondary to COVID-19. This therapeutic approach successfully delayed the need for invasive mechanical ventilation for 48 hours and decreased the mortality rate in all cases.

## Introduction

During the coronavirus disease 2019 (COVID-19) pandemic, the number of patients with bronchospasm requiring mechanical ventilation has increased considerably.

The sudden increase in patients needing intubation represents an unprecedented challenge due to limited availability of equipment in poorly prepared health systems [1] and the lack of evidence to support its long-term benefits. According to Lai, 20.1% of patients with severe acute respiratory syndrome coronavirus 2 (SARS-CoV-2) develop acute respiratory distress syndrome (ARDS) and 9% require invasive mechanical ventilation (IMV) [2]. Poor response to IMV leads to a mortality rate of 10.4% at 24 hours and 61% in the following days in Intensive Care Unit (ICU) stay [3].

The increased mortality in critically ill patients [4], compared with the relatively low mortality rate in the general population [1], has prompted the scientific community to design new therapeutic approaches. Global concern about the pandemic has indeed fueled research for treatments to fight the infection; however, to date, there is no standardized treatment [5] and the process to develop a successful treatment might take months [6].

We presented four cases in which we used early respiratory therapy and pharmacological approach to successfully address acute episodes of bronchospasm delaying the use of invasive mechanical ventilation 48 hours after the acute episode.

## Case presentation

Case 1

A 59-year-old male, COVID-19-positive confirmed with polymerase chain reaction (PCR) assay patient with no significant past medical history came to the Emergency Room (ER) complaining of respiratory distress.

The patient was admitted under suspicion of bronchospasm with preserved acid-base balance [pH: 7.41 (normal range = 7.35-7.45), partial pressure of carbon dioxide (PaCO_2_): 39mmHg (normal range = 33-45mmHg), partial pressure of oxygen (PaO_2_): 55mmHg (normal range = 75-105mmHg)], fraction of inspired oxygen (FiO_2_) of 57 (normal range = >68%), PaO_2_/FiO_2_ was 96 (normal range = >300), and oxygen saturation of 88% associated with SARS-CoV-2 complicated with acute respiratory distress syndrome (ARDS).

**Figure 1:**
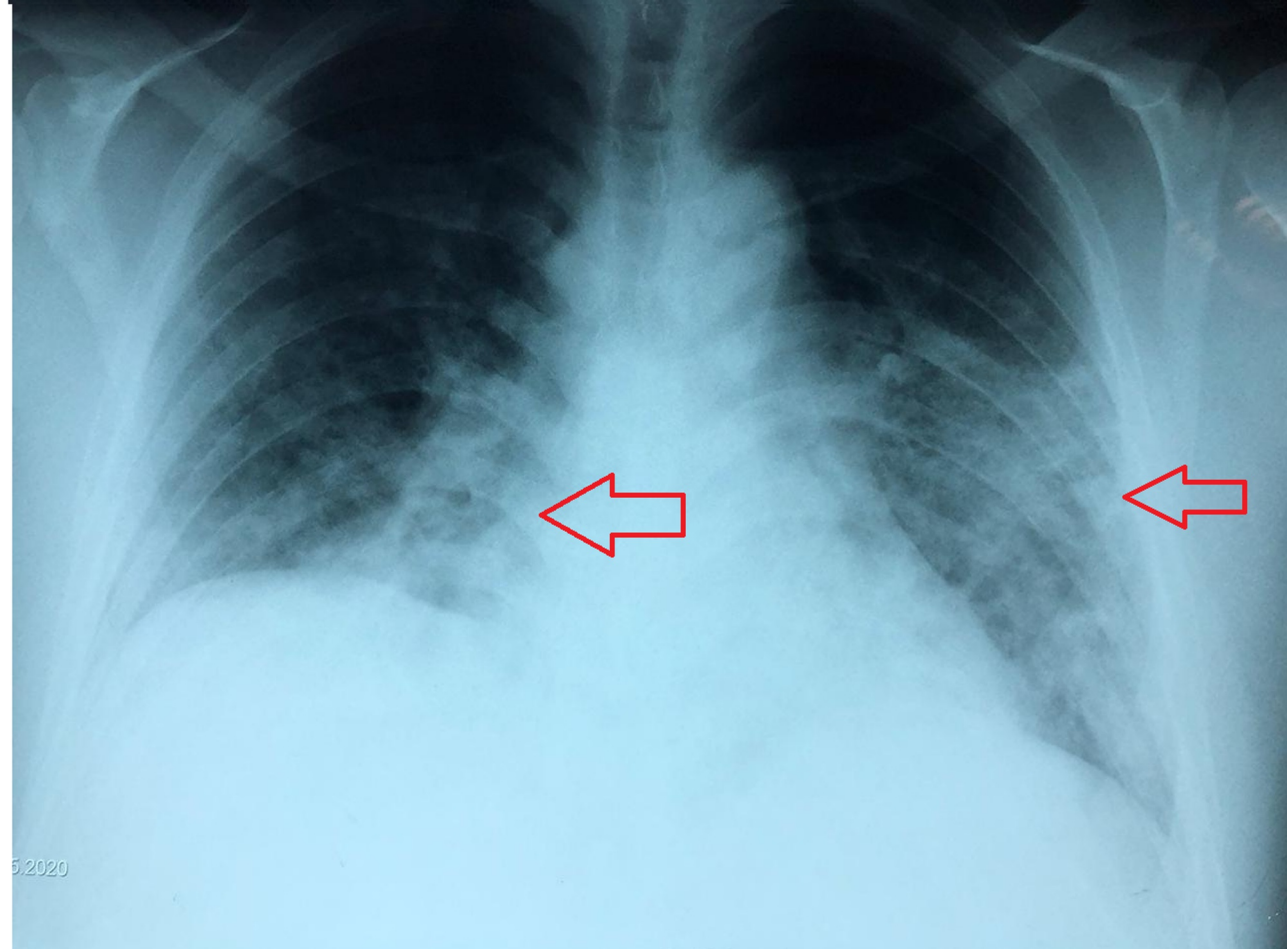
Radiographic assessment of lung involvement Anteroposterior chest X-ray at the time of acute bronchospasm with Radiographic Assessment of Lung Edema (RALE) score 2

On admission, the chest X-ray showed bilateral ground-glass pattern, Radiographic Assessment of Lung Edema (RALE) score 2 (Figure 1) [7]. The patient scored 4 on the Sequential Organ Failure Assessment (SOFA) score and 12 on the Acute Physiology And Chronic Health Evaluation (APACHE) score (Table 1).

**Table 1:** Vital signs and laboratory workup during the acute episode of bronchospasm. MAP: Mean Arterial Blood Pressure. HR: Heart Rate. RF: Respiratory frequency. PaO2: Partial pressure of oxygen. Crea: Creatinine. Hto: Hematocrit. GCS: Glasgow Coma Scale. PaO2/FiO2: arterial oxygen pressure/inspired fraction of oxygen. TB: Total Bilirubin

Temperature (°C)	MAP	HR	RF	PaO2 (mmHg)	pH	Na (mEq/L)	K (mEq/L)	Crea (mg/dL)	Hto (%)	GCS	PaO2/FiO2	Diuresis	TB (mg/dL)	Platelets (×10³/µL)	Vasoactive
37.2	71	85	30	55	7.41	130.2	4.1	0.96	47.8	15	96	850	0.7	233000	NO
36.8	80	91	30	65	7.34	138.7	4.5	1.04	45.4	15	115	1250	0.56	428000	NO
36.2	63	109	47	32	7.26	143.4	4.08	0.56	41.7	15	120	1500	0.49	620000	NO
36.2	65	125	35	71	7.35	145	3.7	0.47	41.6	15	180	1300	0.27	399000	NO
37.1	62	99	29	63	7.39	142.5	4.9	0.58	48	15	104	1250	0.44	296000	NO
36.3	70	102	35	66	7.38	124.2	5.3	0.99	45	15	228	1000	0.61	317000	NO

The initial management comprised placing the patient in the prone position and administering oxygen at high flow through a non-rebreather mask with flow between 10 and 15 liters per minute until reaching 100% FiO_2_. Additionally, respiratory therapy consisting of deep inspiration with an inspiratory hold technique was implemented. Epinephrine and magnesium sulfate were administered as a bronchodilator regimen (Table 2).

**Table 2:** Acute pharmacological treatment for acute bronchospasm

Pharmacological treatment						
Ipratropium bromide			2 puff every 20 minutes for one hour followed by once every 6 hours			
							Adrenalin (1:10000)			0.03 mg intravenously every 8 hours			
Magnesium sulfate			2 gr every 8 hours										

Case 2

A 59-year-old male COVID-19-positive patient with no significant past medical history came to the ER complaining of respiratory distress. The patient was admitted due to suspected bronchospasm associated with SARS-CoV-2 and later diagnosed with ARDS.

**Figure 2:**
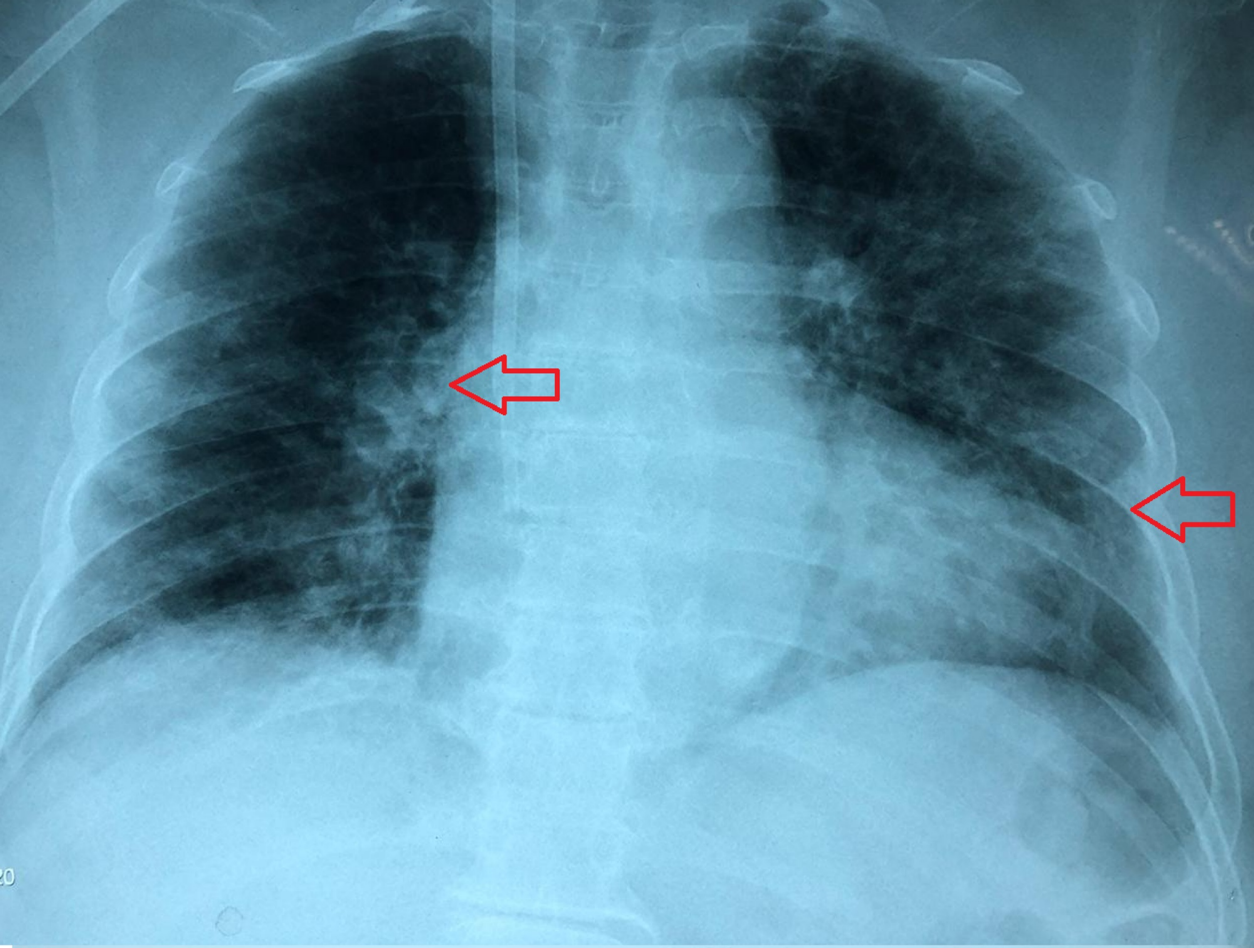
Radiographic assessment of lung involvement Anteroposterior chest X-ray at the time of acute bronchospasm with Radiographic Assessment of Lung Edema (RALE) score 2

On admission, laboratory and imaging tests showed bilateral ground-glass pattern on chest X-ray and a RALE score of 2 (Figure 2) [7]. The arterial blood gases were within normal limits [pH: 7.35 (normal range = 7.35-7.45), PaCO_2_: 44 (normal range = 33-45mmHg), PaO_2_: 58 (normal range = 75-105mmHg)], FiO_2_ of 40 (normal range = >68%), PaO_2_/FiO_2_ was 115 (normal range = >300) and an O_2_ Saturation of 78%. The SOFA score was 3 and APACHE score was 6 (Table 1).

The patient was placed in the prone position and oxygenated at high flow with a non-rebreather mask with flow between 10 and 15 liters per minute until reaching 100% FiO_2_. Additionally, respiratory therapy consisting of deep inspiration with an inspiratory hold technique was started. Epinephrine and magnesium sulfate were administered as a bronchodilator regimen (Table 2).

Case 3

A 33-year-old female patient with morbid obesity was admitted to our hospital due to the risk of COVID-19 related complications.

The patient was managed with antipyretics during hospitalization. On the third day of admission, she developed severe bronchospasm and was transferred to ICU due to poor ventilatory mechanics with preserved acid-base state [pH: 7.35 (normal range = 7.35-7.45), PaCO_2_: 39 (normal range = 33-45mmHg), PaO_2_: 71 (normal range = 75-105mmHg)], FiO_2_ of 50 (normal range = >68%), PaO_2_/FiO_2_ was 182 (normal range = >300) and an O_2_ saturation of 93% associated with SARS-CoV-2. The patient was diagnosed with ARDS.

**Figure 3:**
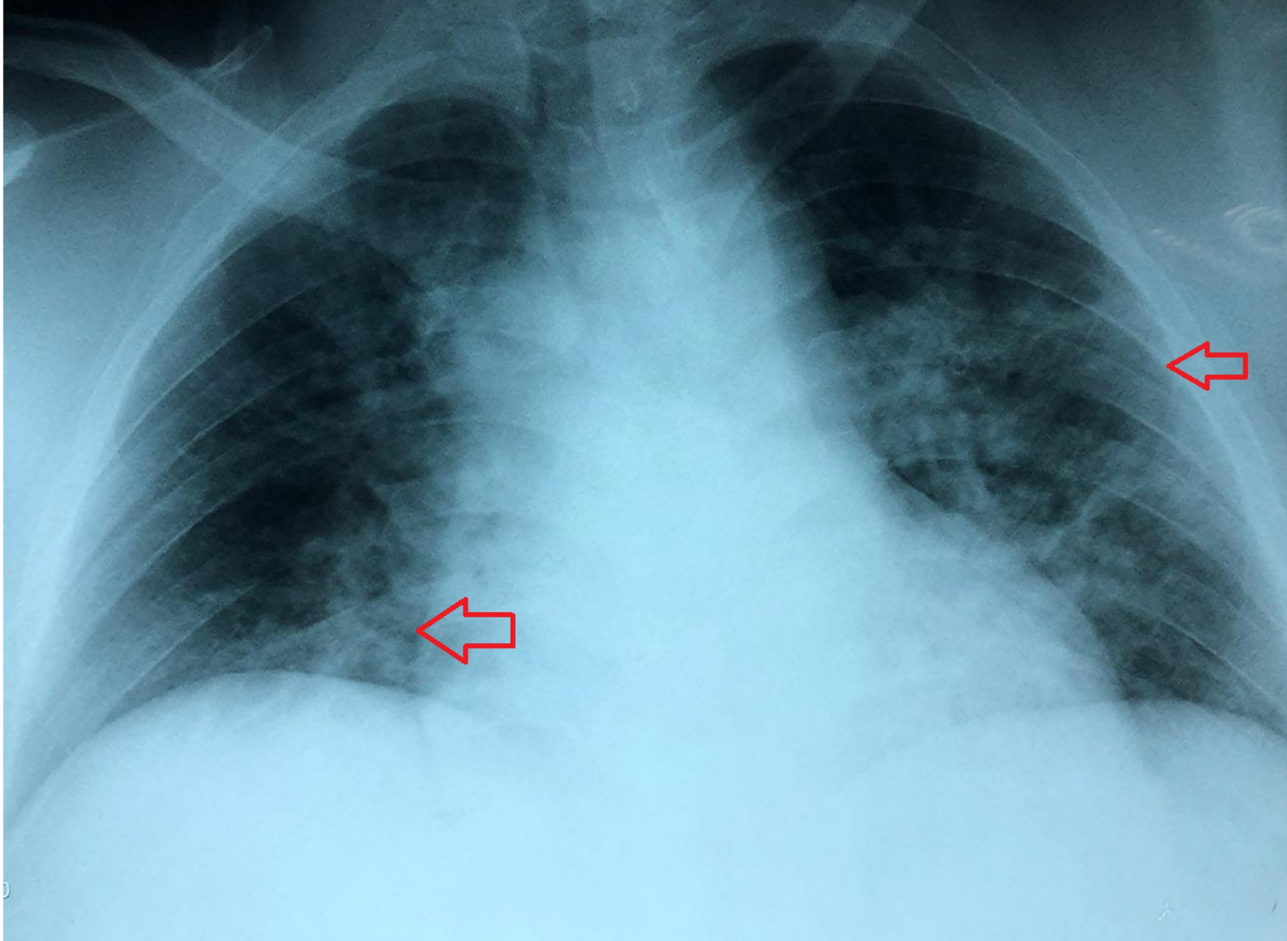
Radiographic assessment of lung involvement Anteroposterior chest X-ray at the time of acute bronchospasm with Radiographic Assessment of Lung Edema (RALE) score 1

The chest X-ray showed bilateral ground-glass pattern, RALE score was 1 (Figure 3) [7]. SOFA score was 3 and APACHE score was 8 (Table 1).

The patient was placed in the prone position and oxygenated at high flow with a non-rebreather mask with flow between 10 and 15 liters per minute until reaching 100% FiO_2_. Additionally, respiratory therapy consisting of deep inspiration with an inspiratory hold technique was started. Epinephrine and magnesium sulfate were administered as a bronchodilator regimen (Table 2).

Case 4

A 39-year-old male COVID-19 positive patient with no significant past medical history came to the ER due to respiratory distress.

Upon admission, the patient underwent laboratory and imaging tests due to the suspicion of SARS-CoV-2 bronchospasm. Arterial blood gases were unremarkable [pH: 7.39 (normal range = 7.35-7.45), PaCO_2_: 31 (normal range = 33-45mmHg), PaO_2_: 63 (normal range = 75-105mmHg)], FiO_2_ of 21 (normal range = >68%), PaO_2_/FiO_2_ was 104 (normal range = >300) and an O_2_ saturation of 89%.

**Figure 4:**
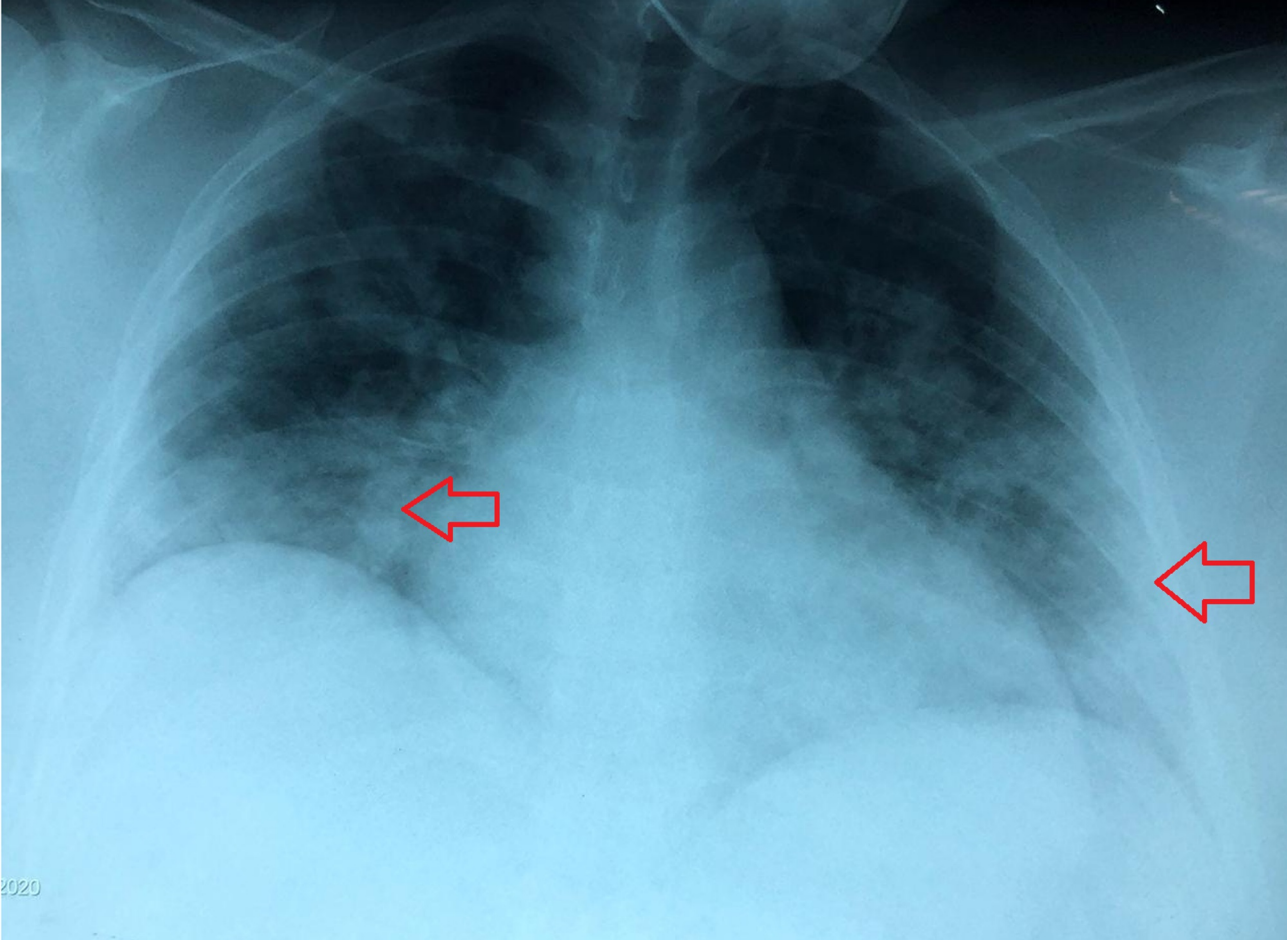
Radiographic assessment of lung involvement Anteroposterior chest X-ray at the time of acute bronchospasm with Radiographic Assessment of Lung Edema (RALE) score 2

Chest X-ray showed bilateral ground-glass pattern, RALE score 2 (Figure 4) [7]. The SOFA score was 3 and APACHE score was 9 (Table 1). The patient was diagnosed with ARDS.

As part of the therapeutic approach, the patient was placed in a prone position with a non-rebreather mask with high flow oxygen between 10 and 15 liters per minute until reaching 100% FiO_2_. Respiratory therapy consisting of deep inspiration with an inspiratory hold technique was started. Also, epinephrine and magnesium sulfate were administered as a bronchodilator regimen (Table 2).

## Discussion

A consensus for acute bronchospasm associated with SARS-CoV-2 infection is the use of mechanical ventilation [8]; however, Zareifopoulos warns of the lack of evidence about the long-term benefits in patients on IMV [4].

To improve ventilatory mechanics and ultimately postpone the need for IMV due to acute bronchospasm in patients diagnosed with COVID-19, we put in place a therapeutic approach consisting of early respiratory therapy and pharmacological bronchospasm rescue approach.

The patients were placed in a prone position during the treatment of the acute episode of bronchospasm. As per, this maneuver could improve PaO_2_/FiO_2_ improving the oxygenation and ultimately postponing the need for mechanical intubation [8]. Theoretically, greater homogeneity in ventilation decreases ventral alveolar distention or dorsal collapse [9].

Concomitantly, early respiratory therapy using deep inspiration with an inspiratory hold technique was initiated. In this technique, the patient is asked to perform a forced expiration followed by deep inspiration which is held for five to seven seconds. The technique was repeated for four cycles of five breaths every hour to improve ventilation and the mobilization of secretions. Although Lazzeri reports the risk of superinfections as a possible complication of respiratory therapy in patients positive for SARS-CoV-2, this event did not occur in our patients [10].

The patients in our study developed their initial episode of severe bronchospasm, based on clinical signs and laboratory tests, at different times of their hospitalization. The events were successfully controlled through the implementation of pharmacologically preventive and rescue therapy for bronchospasm with ipratropium bromide, adrenaline, and magnesium sulfate according to the guidelines for Asthma and Chronic Obstructive Pulmonary Disease (Table 2) [11-13].

Due to the favorable evolution of the patients (20% improvement in respiratory function values in the first four hours compared with the baseline) this therapeutic regime was maintained until the acute presentation resolved. It is worth mentioning that clinical improvement didn’t correlate with radiological improvement.

As all patients showed remarkable improvement in ventilatory effort evidenced by the use of accessory musculature, oxygen saturation, and Kirby index (PaO_2_/FiO_2_), they were discharged from ICU and continued management in the medical floors.

We remark the importance of this case report in the management of patients with severe bronchospasm associated with COVID-19 as the patients who underwent this therapeutic approach didn’t require mechanical ventilation within 72 hours after the acute event. By deferring the use of invasive mechanical ventilation, we have significantly decreased the mortality of patients with ARDS secondary to SARS-CoV-2 admitted to the Intensive Care Unit in our hospital.

## Conclusions

The therapeutic bundle of early respiratory therapy, consisting of deep inspiration with inspiratory hold, and pharmacological bronchospasm rescue decreased the need for invasive mechanical ventilation in patients with bronchospasm associated with SARS-CoV-2 and reduced the mortality rate. We suggest further research studies to standardized this management and implementation of guidelines.

## Appendices

**Table 3:** Vital signs and laboratory workup during the acute episode of bronchospasm. MAP: Mean Arterial Blood PressurE. HR: Heart Rate. RF: Respiratory frequency. PaO2: Partial pressure of oxygen. Crea: Creatinine. Hto: Hematocrit. GCS: Glasgow Coma Scale. PaO2/FiO2: arterial oxygen pressure/inspired fraction of oxygen. TB: Total Bilirubin

Temperature (°C)	MAP	HR	RF	PaO2 (mmHg)	pH	Na (mEq/L)	K (mEq/L)	Crea (mg/dL)	Hto (%)	GCS	PaO_2_/FiO_2_	Diuresis	TB (mg/dL)	Platelets (×10³/µL)	Vasoactive
37.2	71	85	30	55	7.41	130.2	4.1	0.96	47.8	15	96	850	0.7	233000	NO
36.8	80	91	30	65	7.34	138.7	4.5	1.04	45.4	15	115	1250	0.56	428000	NO
36.2	63	109	47	32	7.26	143.4	4.08	0.56	41.7	15	120	1500	0.49	620000	NO
36.2	65	125	35	71	7.35	145	3.7	0.47	41.6	15	180	1300	0.27	399000	NO
37.1	62	99	29	63	7.39	142.5	4.9	0.58	48	15	104	1250	0.44	296000	NO
36.3	70	102	35	66	7.38	124.2	5.3	0.99	45	15	228	1000	0.61	317000	NO

**Table 4:** Acute Pharmacological treatment for acute bronchospasm

Pharmacological treatment						
Ipratropium bromide			2 puff every 20 minutes for one hour followed by once every 6 hours			
							Adrenalin(1:10000)			0.03 mg intravenously every 8 hours			
Magnesium sulfate			2 gr every 8 hours										
